# Neuropeptide Y-mediated sex- and afferent-specific neurotransmissions contribute to sexual dimorphism of baroreflex afferent function

**DOI:** 10.18632/oncotarget.11880

**Published:** 2016-09-07

**Authors:** Yang Liu, Di Wu, Mei-Yu Qu, Jian-Li He, Mei Yuan, Miao Zhao, Jian-Xin Wang, Jian He, Lu-Qi Wang, Xin-Jing Guo, Meng Zuo, Shu-Yang Zhao, Mei-Na Ma, Jun-Nan Li, Weinian Shou, Guo-Fen Qiao, Bai-Yan Li

**Affiliations:** ^1^ Department of Pharmacology, Harbin Medical University, Harbin, China; ^2^ Key Laboratory of Cardiovascular Research of Ministry of Education, Harbin Medical University, Harbin, China; ^3^ Riley Heart Research Center, Division of Pediatric Cardiology, Herman B. Wells Center for Pediatric Research, Department of Pediatrics, Indiana University School of Medicine, Indianapolis, IN, USA

**Keywords:** neuropeptide Y, baroreflex, nodose ganglion, nucleus tractus solitarii, whole-cell patch techniques

## Abstract

**Background:**

Molecular and cellular mechanisms of neuropeptide-Y (NPY)-mediated gender-difference in blood pressure (BP) regulation are largely unknown.

**Methods:**

Baroreceptor sensitivity (BRS) was evaluated by measuring the response of BP to phenylephrine/nitroprusside. Serum NPY concentration was determined using ELISA. The mRNA and protein expression of NPY receptors were assessed in tissue and single-cell by RT-PCR, immunoblot, and immunohistochemistry. NPY was injected into the nodose while arterial pressure was monitored. Electrophysiological recordings were performed on nodose neurons from rats by patch-clamp technique.

**Results:**

The BRS was higher in female than male and ovariectomized rats, while serum NPY concentration was similar among groups. The sex-difference was detected in Y_1_R, not Y_2_R protein expression, however, both were upregulated upon ovariectomy and canceled by estrogen replacement. Immunostaining confirmed Y_1_R and Y_2_R expression in myelinated and unmyelinated afferents. Single-cell PCR demonstrated that Y_1_R expression/distribution was identical between A- and C-types, whereas, expressed level of Y_2_R was ∼15 and ∼7 folds higher in Ah- and C-types than A-types despite similar distribution. Activation of Y_1_R in nodose elevated BP, while activation of Y_2_R did the opposite. Activation of Y_1_R did not alter action potential duration (APD) of A-types, but activation of Y_2_R- and Y_1_R/Y_2_R in Ah- and C-types frequency-dependently prolonged APD. N-type *I*_Ca_ was reduced in A-, Ah- and C-types when either Y_1_R, Y_2_R, or both were activated. The sex-difference in Y_1_R expression was also observed in NTS.

**Conclusions:**

Sex- and afferent-specific expression of Neuropeptide-Y receptors in baroreflex afferent pathway may contribute to sexual-dimorphic neurocontrol of BP regulation.

## INTRODUCTION

The pressor responses induced by neuropeptide Y (NPY) are greater in males compared with age-matched females [1] and the underlying molecular and cellular mechanisms are complex and largely unknown. Although no difference in serum concentration of NPY was found between genders, it could be elevated in both hypertensive men and women [2], suggesting at least that NPY itself would not be responsible for gender-related difference in blood pressure (BP) under physiological condition and the sex-differential expression of NPY receptors would be highly expected in either peripheral or central site of BP regulation. Early studies have demonstrated that activation of type-I NPY receptor (Y_1_R) leads to a vasodepressor response [3, 4], while type-II NPY receptor (Y_2_R) activation induces vasopressor action in nucleus tractus solitarii (NTS) [4, 5], indicating that Y_1_R and Y_2_R activations often mediate an opposite pressor response. Several lines of evidence also imply the central mechanisms of NPY in BP regulation and potentially differential role of its receptor activation at different level of baroreflex afferent pathway, such as nodose ganglion (NG) and NTS. Firstly, significant effects of gender on the central actions of NPY on vasopressin and BP have been reported [6]; secondly, Y_2_R mRNA expression is dramatically increased in the NTS at hypertensive condition, whereas it is decreased in the NG under the same experimental condition [7], suggesting that NPY and its receptors participate in the BP regulation under both physiological and hypertensive condition via modulating baroreflex afferent function. Recent results have indicated that naturally occurring genetic variation at the Y_1_R locus has implications for heritable autonomic control of the circulation and hypertension, suggesting novel pathophysiological links among the Y_1_R locus, autonomic activity, and BP [8]. Y_2_R expression is upregulated in spontaneously hypertensive rats [9] and endogenous expression of Y_2_R is also documented in neuroendocrine cells and neuroendocrine tissues including the brainstem of a rodent model of hypertension [10].

Collectively, all published records indicate that NPY plays a pivotal role in BP regulation and development of hypertension through either peripheral or central pathway with gender-specific manner. However, there is no published record showing the sex-specific expression and distribution of Y_1_R and Y_2_R in baroreflex afferent pathway including NG and NTS under normal or hypertensive condition. Moreover, a low-threshold and sex-specific distribution of myelinated Ah-type baroreceptor neurons (BRNs) housed in NG and NTS has been identified [11–14]. The neuroexcitability of this subpopulation depends upon the presence of estrogen (17β-E_2_) [15–17] and is regulated by neurotransmitter [18], which may impact on the sexual dimorphism of baroreflex afferent function and neurocontrol of circulation [19]. Therefore, this study aims to explore sex- and afferent-specific expression and distribution of Y_1_R and Y_2_R in NG and NTS at tissue and single-cell level, the effect of direct injection of Y_1_R or Y_2_R agonist into NG on the mean arterial pressure (MAP), and the ion channel mechanism of neuroexcitation induced by Y_1_R and Y_2_R activation.

## RESULTS

### Estrogen-dependent changes in baroreceptor sensitivity

**Figure 1:**
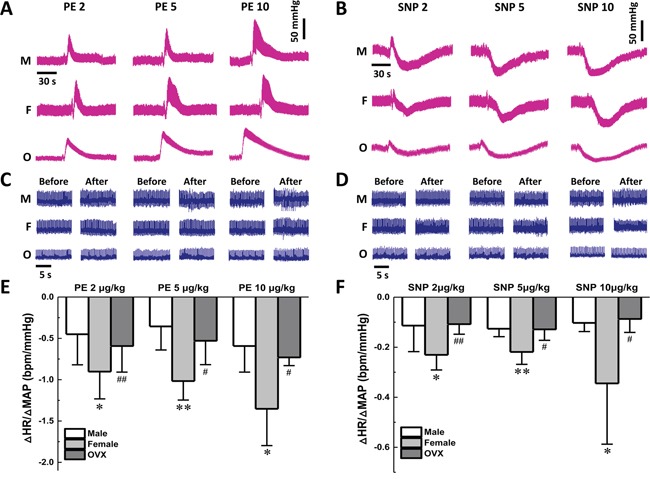
Effect on baroreflex sensitivity of gender difference during vasoactive drugs application Femoral artery catheterization was applied to measure the change of MAP and venous cannula was used for administration of PE and SNP. **A-B.** The representative recordings of MAP collected from male (M; *n* = 7), female (F; *n* = 7), and ovariectomized (OVX; *n* = 4) rats in the presence of 2, 5, and 10 μg/kg of PE and SNP, respectively. **C-D.** The representative recordings of the heart rate (HR) along with the blood pressure (BP) changes; **E-F.** The summarized changes of BRS (ΔHR/ΔMABP, bpm/mmHg) when treated with PE and SNP at different concentration in each group. The averaged data were expressed as means ± SD. *P < 0.05 and **P < 0.01 *vs.* Male group; ^#^*P* < 0.05 and ^##^*P* < 0.01 *vs.* Female group. Scale bars were applied for all recordings.

To explore whether the depressor reflex modulation of blood pressure is female hormone-dependent, the baroreceptor sensitivity (BRS) was tested in adult males, age-matched females, as well as ovariectomized (OVX) female rats by measuring the mean arterial pressure (MAP) in the presence of phenylephrine (PE) or sodium nitroprusside (SNP) (2, 5, and 10 μg/kg; Figure 1A & 1B). Meanwhile, electrocardiogram (ECG) was monitored accordingly (Figure 1C & 1D). The results showed that the values of ΔHR/ΔMABP, an index of BRS, were dose-dependently increased in females than that in males, and reversed completely back to the level of males in the OVX rats (Figure 1E & 1F). This observation suggests that sex hormones may affect the function of catecholamines. The neuropeptide-Y (NPY), as a neurotransmitter and potent vasoconstrictor, influences sympathetic activation together with others including norepinephrine or angiotensin-II [20]. In this regard, serum concentration of NPY was detected by ELISA, and no significant difference was observed between males and age-matched females (276.5 ± 144.9 *vs*. 266.4 ± 125.3 pg/ml, *P* > 0.05, *n* = 10). In addition, surgical removing of the ovaries did not affect serum NPY content (279.5 ± 98.6 pg/ml *vs*. either males or females, *n* = 8). These data suggested that serum NPY itself may not be the causal factor for the different baroreflex afferent function of males and female.

### Sex-specific and/or -estrogen (17β-E_2_)-dependent expression of Y_1_R and Y_2_R in nodose ganglia

**Figure 2:**
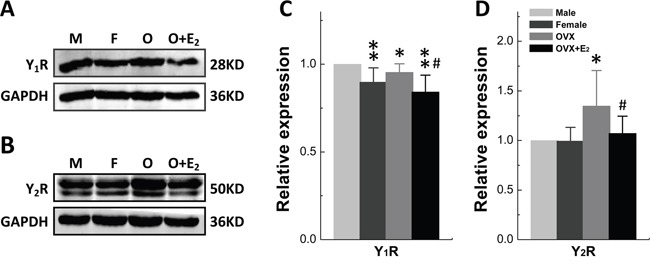
Gender difference in protein expression of Y_1_R and Y_2_R in Nodose Ganglia Protein was accessed in nodose ganglia of adult male (M), aged-matched female (F), ovariectomized (OVX) female rats and OVX administrated 17β-estradiol (17β-E_2_). **A** and **B.** Protein bands for Y_1_R and Y_2_R, respectively; **C** and **D.** Averaged data of relative expression profiles for Y_1_R and Y_2_R. The averaged data were presented as mean ± SD. *n* = 4 duplicated tests in which the tissue was collected from 10 rats of each group. *P < 0.05 and **P < 0.01 *vs*. male, ^#^*P* < 0.05 *vs*. OVX.

We then tested if there is any difference in the expression and distribution of NPY receptor between males and females. The protein expression of Y_1_R and Y_2_R was assessed in nodose ganglia (NG). The results showed that Y_1_R expression was lower (*P* < 0.01) in females compared with age-matched males, which was slightly but not significantly upregulated by ovariectomy (*P* = 0.116 *vs*. female). Nevertheless, Y_1_R was remarkably down-regulated by 17β-E_2_ treatment (*P* < 0.01 or *P* < 0.05 *vs*. male or OVX) (Figure 2A and 2C). Notably, the expression of Y_2_R was dramatically enhanced by OVX (*P* < 0.05 *vs*. either male or female) and reversed (*P* < 0.05 *vs*. OVX) by 17β-E_2_ treatment (Figure 2B and 2D) even though the expression level was identical between male and female rats. These observations suggest that the protein expression of both Y_1_R and Y_2_R are in a sex-specific or estrogen-dependent manner in the NG.

### Immunohistochemical analysis of Y_1_R and Y_2_R at tissue of nodose ganglia

**Figure 3:**
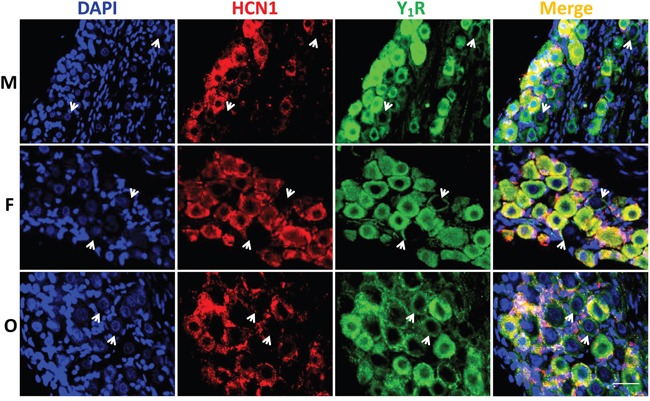
Immunohistochemical staining for Y_1_R The Y_1_R staining was performed in nodose ganglia from male (M, top), female (F, central), and ovariectomized (O, bottom) rats. The nucleus, hyperpolarization-activated channel specifically expressed on myelinated afferents (HCN1-positive), and Y_1_R were labeled by the antibodies against DAPI (blue), HCN1 (red), and Y_1_R (green). Arrowheads: indicate the neurons with unmyelinated afferents (HCN1-negative). The scale bar: 50 μm.

**Figure 4:**
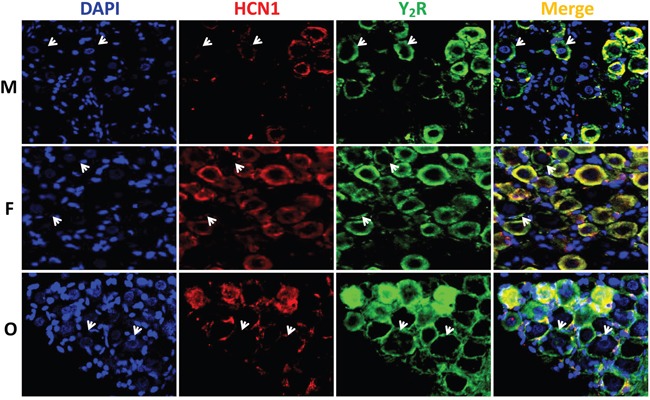
Immunohistochemical staining for Y_2_R The Y_2_R staining was performed in nodose ganglia from male (M, top), female (F, central), and ovariectomized (O, bottom) rats. The nucleus, hyperpolarization-activated channel specifically expressed on myelinated afferents (HCN1-positive), and Y_2_R were labeled by the antibodies against DAPI (blue), HCN1 (red), and Y_2_R (green). Arrowheads: indicate the neurons with unmyelinated afferents (HCN1-negative). The scale bar: 50 μm.

To further confirm the expression of NPY receptors in nodose ganglion, the immunohistochemical staining was carried out. Both Y_1_R (Figure 3) and Y_2_R (Figure 4) were detected in the cell-membrane and cytoplasm of myelinated afferents (HCN1-positive), whereas they were only detected in the cell-membrane of unmyelinated afferents (HCN1-nagetive, indicated as white arrowheads). Quantification analysis (Supplementary Table S1) showed that, for Y_1_R/HCN1-positive, the fluorescent intensity was lower in female (*P* < 0.05 vs. male), which was further downregulated by OVX (*P* < 0.01 *vs*. female). There was no difference between males and age-matched females in Y_2_R/HCN1-positive, OVX dramatically upregulated Y_2_R level (*P* < 0.01 *vs*. female). In the case of HCN1-negative populations, the difference in fluorescent intensity for Y_1_R was not established among groups. However, the intensity for Y_2_R of females was higher (*P* < 0.05 *vs*. male) and completely reversed by OVX (*P* <0.01 *vs*. female). Even though the averaged results of fluorescent analysis do not completely match with molecular observations, the difference might be explained by the YR whole tissue detection in molecular analysis and the afferent-specific quantification in fluorescence. The YR expression in cells other than neurons such as the satellite cells around the neurons (as indicated by the pink area of merged images from Figure 3 and 4) in the tissue of NG may also significantly influence the final analysis.

### Afferent-specific distribution of Y_1_R and Y_2_R in identified single BRNs from female rats

**Figure 5:**
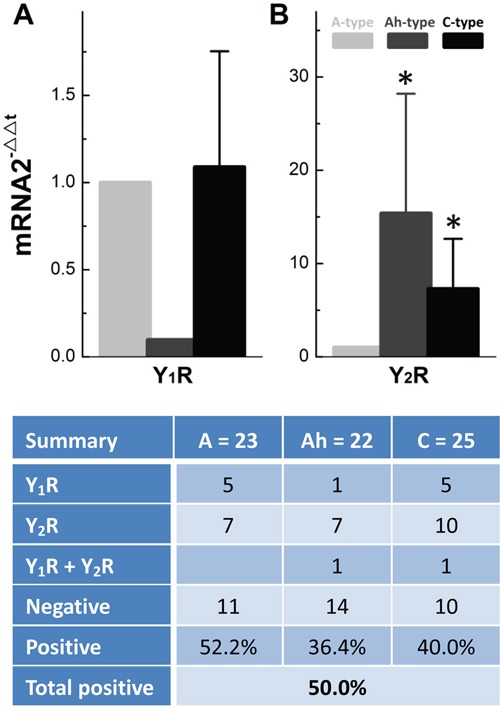
Cell-specific expression and distribution of mRNA of Y_1_R and Y_2_R in identified single BRNs from adult female rats The action potential (AP) was collected under the current-clamp mode of whole-cell configuration and the afferent fiber types of BRNs was identified by standard electrophysiological validation, which was then collected for single-cell RT-PCR. **A.** Relative mRNA expression of Y_1_R; **B.** Relative mRNA expression of Y_2_R; *n* = 22-25. *P < 0.05 *vs*. A-type BRNs. The bottom table: the percentage distribution of Y_1_R and Y_2_R in each category neurons.

To determine the afferent-specific expression, single-cell RT-PCR was employed in identified single BRNs. The data (Figure 5A) showed that Y_1_R mRNA equally expressed and distributed (5/23 or 5/25) in A- and C-types, whereas very low expression level of Y_1_R was found in only 1 of 22 tested Ah-type BRNs (1/22, Figure 5A & bottom tab.), indicating almost no Y_1_R expression in Ah-types. However, Ah- and C-types BRNs expressed more than 15 and 7 folds (*P* < 0.05 *vs*. A-type) of Y_2_R (Figure 5B), respectively, even though the distribution in the number of positive detections was identical among A- (n = 7/23, 30.4%), Ah- (n = 7/22, 31.8%), and C-types (10/25, 40%), suggesting a predominant role of Y_2_R in the function of Ah- and C-type BRNs (Figure 5 bottom tab.).

### Y_1_R and Y_2_R activation-mediated changes in blood pressure by nodose ganglion injection

**Figure 6:**
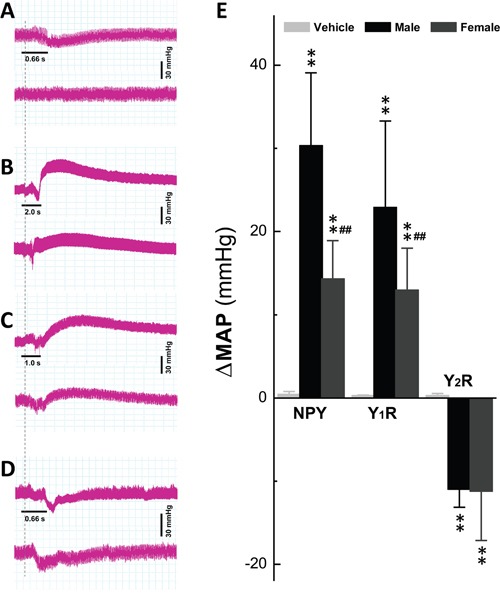
Y_1_R and Y_2_R activation-mediated changes in blood pressure by NG microinjection The left side of nodose ganglion (NG) and Vagus were dissected and exposed carefully on anesthetic rats. The femoral artery cannulation was performed and the blood pressure (BP) was collected before and after administration of 5 μg NPY, Pro-34, and NPY13-36, respectively. **A.** the representatives of BP recordings before and after saline (top) and NPY placed on the surface (bottom) of NG; **B–D.** representative of BP recordings before and after NPY, Pro-34, and NPY13-36, respectively, in male (top) and female rats (bottom); The dash line indicates the time of the beginning of treatment. **E.** the summarized changes in the net mean arterial pressure (ΔMAP) before and after each treatment in male (*n* = 6) and female (*n* = 6) rats. The averaged data were expressed by mean ± SD. **P < 0.01 *vs*. vehicle control, ^##^*P* < 0.01 *vs*. male group.

We then tested if Y_1_R and Y_2_R activation may produce opposite effects in BP regulation. The changes in mean arterial pressure (MAP) were investigated when NPY and selective agonists of Y_1_R, Pro-34 and Y_2_R, NPY13-36 were directly injected into NG (Figure 6). The results showed that both saline and 5 μg of NPY placed right on the surface of NG did not induce significant changes in BP (Figure 6A top and bottom). However, in male rats, 5 μg NPY and Pro-34 elevated BP dramatically (Figure 6B and 6C, top; *P* < 0.01 *vs*. control), whereas 5 μg NPY-13-36 decreased BP (Figure 6D, top; *P* < 0.01 vs. control). Most importantly, the averaged data (Figure 6E) showed that Y_1_R-mediated BP elevations were stronger (Figure 6B & 6C, bottom tab., *P* < 0.01) compared with females with either NPY or Pro-34, suggesting Y_1_R activation-mediated BP upregulation at the level of NG. Intriguingly, the sex-difference in Y_2_R-mediated reduction of BP was not conformed and the effect of Y_1_R was much stronger than that of Y_2_R, suggesting that Y_1_R and Y_2_R activation play an opposite action in BP regulation at the 1^st^-order neurotransmission of baroreflex afferent pathway, and NPY-mediated upregulation of BP by Y_1_R stimulation presumably masks BP downregulation due to its Y_2_R activation.

### Y_1_R or Y_2_R-mediated similar down-regulation of neuromodulation in myelinated A-type BRNs by inhibition of presynaptic Ca^2+^ channel

**Figure 7:**
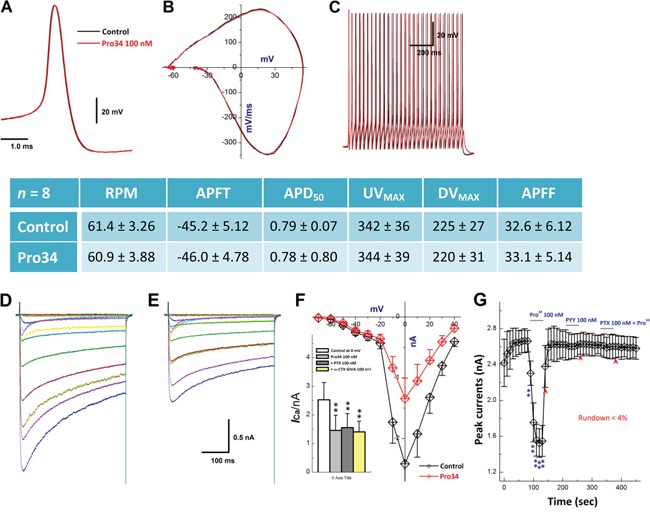
Effects of Pro34 on AP discharge profiles and *I*_Ca_ in identified A-type BRNs **A–B.** action potential (AP) and derivative changes before and after 100 nM Pro34; **C.** repetitive discharge before and after Pro34; Center table: summarized changes in AP discharge profiles; **D–E.** the Ca^2+^ current (*I*_Ca_) in identified A-BRNs using slice preparation before and after Pro34; **F.** current-voltage relationship (I-V) of *I*_Ca_ before and after Pro34, ***inset***: averaged data of I-V with different treatments, *n* = 6-7, **P < 0.01 *vs*. control at 0 mV; **G.** time course of *I*_Ca_ alternations in the presence of Pro34, 300 nM BIBP3226 (Y_1_R antagonist) + Pro34, and 100 nM pertussis toxin (PTX) + Pro34, respectively, *n* = 5 complete recordings, *P < 0.05 and **P < 0.01 *vs*. before. Scale bars in (E) also apply for (D).

Upon the expression profile for Y_1_R in A-type BRNs, the effect of Y_1_R activation on action potential (AP) trajectory and N-type calcium currents (*I*_Ca_) was investigated. Firstly, Y_1_R activation by Pro34 (100 nM), Y_1_R selective agonist, showed no effects on AP waveshape and discharge profiles (Figure 7A-7C) but significantly reduced current density of *I*_Ca_ with equal efficacy of 300 nM ω-CTX. The current was completely blocked by BIBP3226 (300 nM), a Y_1_R selective antagonist and PTX 100 nM, the blocker for G-protein coupled receptor, respectively (Figure 7D-7G). Even though AP discharge was not changed in the presence of Pro34, Y_1_R activation-mediated reduction in current density of *I*_Ca_ may still change the neurotransmission in NTS due perhaps to the similar membrane structure between soma and its pre-synapse [21]. Similar results were also observed by Y_2_R activation in separate set of A-type BRNs under the same experimental condition (Data not shown). This phenomenon may attribute to lacking of the co-localization between KCa1.1 and N-type Ca^2+^ channels in myelinated A-type cells even though the expression of theses channels could be identified.

### Y_2_R-mediated peripheral and integrations in sex-specific and low-threshold myelinated Ah-type BRNs by presynaptic BK-KCa inactivation

**Figure 8:**
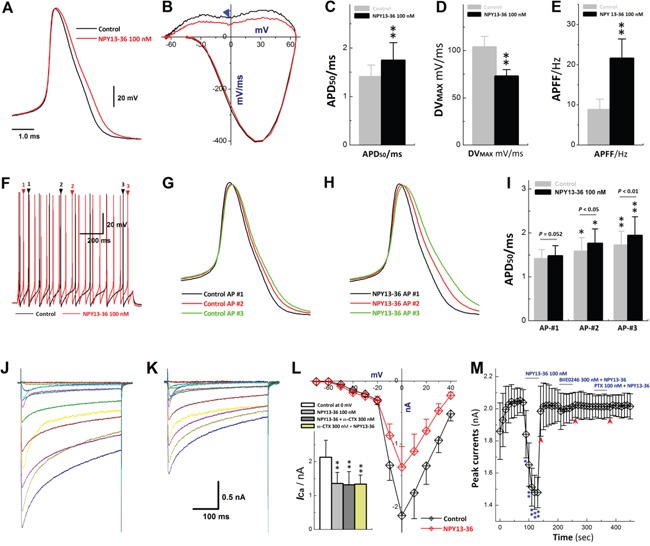
Effects of NPY13-36 on AP discharge profiles and *I*_Ca_ in identified Ah-type BRNs **A–B.** action potential (AP) and derivative changes before and after 100 nM NPY13-36; **C–E.** summarized changes in APD_50_, DV_MAX_, and APFF in the presence of NPY13-36, *n* = 6-10, **P < 0.01 *vs*. control; **F.** repetitive discharge before and after NPY13-36; **G–H.** frequency-dependent prolongation of APD_50_ by superimposition of APs pointed by arrows with numbers during repetitive firings before and after NPY13-36; **I.** averaged data for frequency-dependent prolongation, *n* = 7, *P < 0.05 and **P < 0.01 *vs*. control; **J–K.** whole-cell Ca^2+^ currents (*I*_Ca_) recorded in Ah-types identified by the conduction velocity (CV) using slice preparation before and after NPY13-36; **L.** current-voltage relationship (I-V curve) of *I*_Ca_ before and after NPY13-36, ***inset***: averaged data of I-V with different treatments, *n* = 5-7, **P < 0.01 *vs*. control at 0 mV; **M.** time course of *I*_Ca_ alternations in the presence of NPY13-36, 300 nM BIIE0246 (Y_2_R antagonist) + NPY13-36, and 100 nM pertussis toxin (PTX) + NPY13-36, respectively, *n* = 5 complete recordings, *P < 0.05 and **P < 0.01 *vs*. control. Scale bars in (A) also apply for (G-H); scale bar in (K) also apply for (J).

Compared with A-types, a presumed leading role of Y_2_R in sexual dimorphism in BRx afferent function is expected considering its extremely higher expression in low-threshold and sex-specific myelinated Ah-type BRNs (Figure 5). In electrophysiological identified Ah-types (Figure 7A-7B), Y_2_R activation by NPY13-36 (100 nM), a selective Y_2_R agonist, markedly prolonged AP duration (APD_50_) and slowed the maximal downstroke velocity (DV_MAX_) with increase in AP firing frequency (APFF) (Figure 8A-8F), notably broadened the frequency-dependent APD (Figure 8G-8I) and inhibited N-type *I*_Ca_ (Figure 8J-8M). These data imply that the increased APD may allow more presynaptic Ca^2+^ influx and lead to more neurotransmitter release. This hypothesis seems reasonable, but Y_2_R activation caused *I*_Ca_ inhibition may also directly reduce the Ca^2+^ influx at presynaptic membrane leading to an opposite action on neurotransmission. Therefore, additional investigations would definitely be necessary.

### Y_1_R- and Y_2_R-mediated similar neuromodulation in unmyelinated baroreceptor afferents

**Figure 9:**
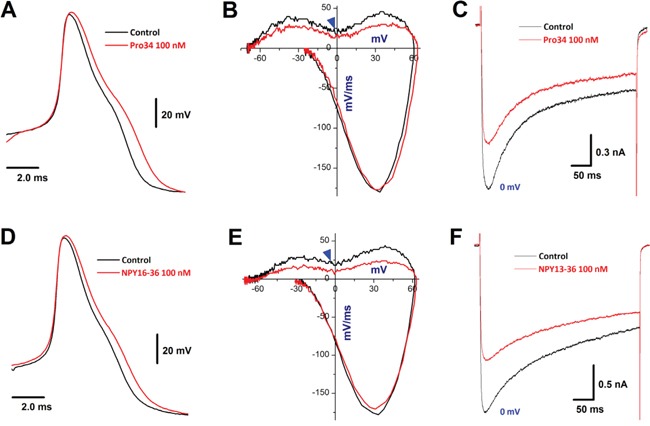
Effects of directly Y_1_R and Y_2_R stimulation on AP and *I*_Ca_ in identified C-type BRNs **A–B.** action potential (AP) and derivatives before and after Y_1_R (100 nM Pro34); **C.** representative recordings of Ca^2+^ currents (*I*_Ca_) at 0 mV before and after 100 nM Pro34; **D–E.** AP and derivatives before and after Y_2_R activation (100 nM NPY13-36); **F.** representative recordings of *I*_Ca_ at 0 mV before and after 100 nM NPY13-36. The center of repolarization hump is indicated by (▼).

In electrophysiological identified unmyelinated C-type BRNs, Y_1_R (Figure 9A-9C) and Y_2_R (Figure 9D-9F) activation caused a similar APD_50_ prolongation and total inward current reduction as revealed by displacement current of phase plots with the decrease in the current density of *I*_Ca_. In the presence of Pro34 and NPY16-36, the APFF was increased with an activity-dependent AP broadening. (Supplementary Table S2).

### Sexual- and estrogen (17β-E_2_)-dependent expression of Y_1_R, rather than Y_2_R in NTS

**Figure 10:**
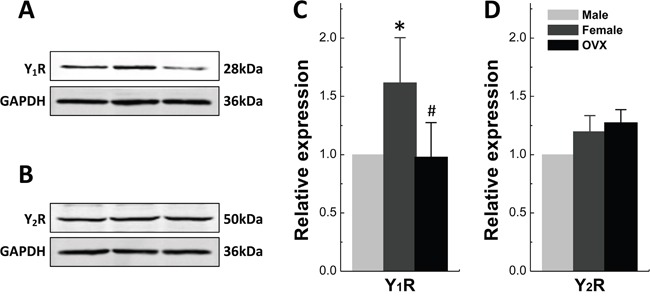
Protein Expression of Y_1_R and Y_2_R in Nucleus of Tractus Solitarii Protein expression was accessed in tissue of nucleus of tractus solitarii (NTS) collected from adult male, aged-matched female, ovariectomized (OVX) female rats. **A** and **B.** Protein bands for Y_1_R and Y_2_R, respectively; **C** and **D.** Averaged data of relative expression profiles for Y_1_R and Y_2_R. The averaged data were presented as mean ± SD. *n* = 4 duplicated tests in which the tissue was collected from 6 rats of each group. *P < 0.05 *vs*. male, ^#^*P* < 0.05 *vs*. female.

The NTS is the center converged visceral afferent inputs from Vagus and aortic depressor nerve (ADN) relayed at the NG. The clarification of the expression and distribution of Y_1_R and Y_2_R in NTS would be critical for fully understanding of NPY-mediated sexual dimorphism in neurocontrol of circulation. To answer this question, the tissue of NTS were collected from adult male, age-matched female, and OVX rats for the immunoblot study. The data (Figure 10) indicated that the expression of Y_1_R, rather than Y_2_R is sex-specific and estrogen-dependent. Y_1_R expression in females was 161.7% (*P* < 0.05) compared with male group, and was completely downregulated in OVX to the equivalent level to males (Figure 10C). The mRNA expressions of Y_1_R and Y_2_R were also tested and identical expression pattern was observed (data not shown).

## DISCUSSION

The major contribution of the current investigation is to demonstrate for the first time that sex- and afferent-specific expression and distribution of Y_1_R and Y_2_R are observed in baroreflex afferent pathway including NG and NTS by the use of immunoblotting and immunohistochemistry, as well as single-cell RT-PCR technique in identified baroreceptor neurons. Additionally, activation of Y_1_R and Y_2_R mediate differential neuroexcitation and Ca^2+^ channel modulation in myelinated A-, Ah- and unmyelinated C-type BRNs identified by electrophysiological validations. These results suggest that NPY and its receptor system play a crucial role in sexual dimorphism of BRx afferent function and neurocontrol of BP regulation.

Increasing body of evidence has demonstrated that NPY receptor expresses in both CNS [22] and PNS [23] and is involved in gender-mediated regulation of BP [1] and hypertension [7]. Previous researches [24, 25] have suggested that NPY is co-stored and co-released with norepinephrine (NE) and other catecholamines in adrenal medulla or from the postganglionic sympathetic nerves to influence the cardiovascular system and correlates with sympathetic activation [26]. However, they do not always have the synergistic action to influence hemodynamic effects. Especially in coronary and cerebral vessels, NPY induces significantly vasoconstriction where NE is not effective [27, 28]. Our present data demonstrated a dramatic sex- and estrogen-related difference in BRS with identical serum concentration of NPY among male, age-matched female, and ovariectomized female rats. This observation implied that NPY may influence BRx afferent function by the differential expression of its receptor subtypes in NG and NTS. The present study indicated that the sex-specific lower expression of Y_1_R in females and the upregulation by lacking of estrogen at NG level, which may explain at least partially why the BP is lower in females *vs*. age-matched males and significantly increased by OVX procedure observed in our previous observation [29]. These data are also consistent with the notion that Y_1_R mediates significant sympathetic vasoconstriction [30, 31]. Although the Y_2_R expression is not sex-specific, it was upregulated by OVX procedure and downregulated by estrogen treatment. Considering that Y_1_R and Y_2_R often mediate an opposite response of MAP in the present study (Figure 6) and work from others [5], the peripheral compensatory mechanism may exist to counteract the Y_1_R-mediated vasoconstriction and elevated BP in OVX via overexpression of Y_2_R. Upregulation of Y_2_R has also been confirmed in the rat model of heart failure [32] and may be well explained by the evidence of parasympathetic vasodilation through presynaptic expression of Y_2_R.

The sex-difference in Y_1_R and Y_2_R expressions has been confirmed in the tissue level of NG. However, the afferent-specific expression of these receptors needs to be clarified to fully understand the cellular mechanism of NPY. Due to the multiple afferent neuron types, the single-cell RT-PCR [29, 33] would be the best to detect mRNA expression in electrophysiologically identified individual neurons [13]. The result has demonstrated the positive detection for A-, Ah, and C-BRNs are 52.2%, 36.4%, and 60%, respectively; and the ratios of Y_1_R/Y_2_R for A-, Ah, and C-BRNs are 5/7, 1/7, and 5/10 as well. Interestingly, Y_2_R not only expresses at higher level but also distributes predominantly in low-threshold and sex-specific subpopulation of Ah-BRNs, suggesting a dominant role of Ah-BRNs in sexual-dimorphism of BRx afferent function. Even though Y_1_R equally expresses in A- or C-BRNs, its expression level is only about 1/10th in Ah-BRNs. In stark contrast, the expression level for Y_2_R is more than 15 or 7 folds higher in Ah-BRNs than that in A- or C-BRNs. This novel finding for the first time demonstrated that the afferent-specific expression profiles of Y_1_R and Y_2_R and the likely role of Y_1_R and Y_2_R in the neurocontrol of circulation and BP regulation at the cellular and molecular levels of BRx afferent pathway.

From the functional point of view, Y_1_R or Y_2_R activation-mediated neuroexcitation and underlying ion channel mechanism are the further questions to be answered. For A-type BRNs, Y_1_R activation did not alter the AP trajectory but significantly decreased the *I*_Ca_ density, suggesting that N-type *I*_Ca_ is not involved in the formation of AP waveform [34] or the coupling between KCa1.1 [35] and N-type Ca^2+^ channel although both KCa1.1 α- and β4-subunits were identified [36]. This observation suggests that NPY may be not critical for the neuroexcitation of A-types but play some role in cell signaling through *I*_Ca_ modulation. Whereas, for C-type BRNs, due to the significant higher expression of Y_2_R observed by single-cell data and large number of its population compared with A-BRNs, somewhat important roles in sex-specific neuromodulation at BRx afferent pathway would be expected.

Since the sex-specific distribution [12], the key role in BRx afferent function [19], unique higher expression of Y_2_R from the current observation, and the effect of Y_1_R or Y_2_R activation on the neuroexcitation of Ah-type BRNs would be the key explanation for the sex-dimorphic BRx. Therefore, by Y_2_R activation, the AP repolarization was significantly altered with longer APD_50_, slower DV_MAX_, faster APFF, and lesser total outward K^+^ currents from AP waveform and phase plots, respectively. Interestingly, activity- or frequency-dependent AP broadening [11, 37] was further enhanced in Ah-type BRNs by Y_2_R activation, rather than Y1R stimulation (data not shown), strongly suggesting the Y_2_R activation-mediated KCa1.1 inactivation [35, 36] indirectly due to N-type Ca^2+^ channel inhibition through the coupling mechanism.

**Figure 11:**
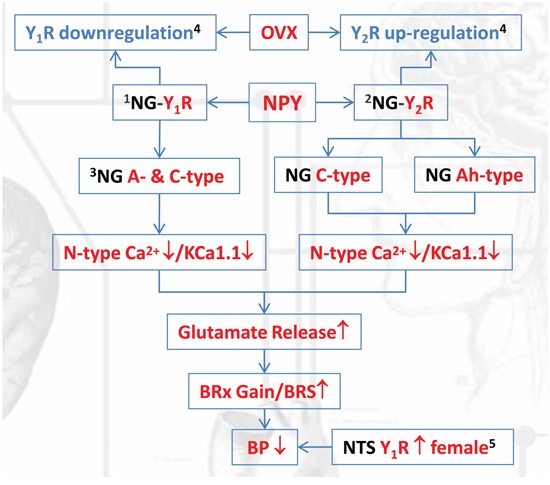
The schematic diagram regarding the cellular mechanism underlying neuropeptide Y-mediated sex- and afferent-specific neurotransmissions of blood pressure regulation Notes for the superscript: (1). Y_1_R highly expressed in myelinated A-type BRNs; (2). Y_2_R mainly expressed in myelinated Ah-type BRNs; (3). action potential duration (APD) was not altered by N-type Ca^2+^ channel inhibition but KCa1.1; (4). This action was restored by estrogen treatment; (5). Y_1_R expression in nucleus tractus solitarii (NTS) was opposite to that in nodose ganglia (NG).

Even though intriguing observations from the cardiovascular literature have provided quantitative evidence that myelinated and unmyelinated cardiovascular afferents evoke not only different frequency-dependent reflex responses but also potential and distinctly different sensory information processing mechanisms [38, 39]. These differences could be explained at least partially by the sex- and afferent-specific expression of Y_1_R and Y_2_R and an opposite action of Y_1_R and Y_2_R activation in blood pressure regulation. Additionally, the difference in peripheral and central mechanism in neurocontrol of circulation mediated by NPY has been identified [3, 5, 20], manifested as a hypotensive and hypertensive responses by Y_1_R and Y_2_R activation in NTS (central), which was a stark contrast compared with by Y_1_R and Y_2_R activation in NG (peripheral). Even though the central hypotensive action of NPY is led by Y_1_R activation [20], the sex-dimorphism in NPY receptor expression is not elucidated so far in NTS. Apparently, averaged Y_1_R protein expression is markedly higher in females than that in age-matched male rats, which is downregulated by OVX, and similar expression pattern for Y_2_R is detected in NTS. This result may contribute, at least in part, to the sex-difference in BP of animal [19, 29] and human [40] with an estrogen-dependent fashion. Moreover, we have a strong reason to believe that myelinated Ah-type barosensitive neurons housed in NTS [41, 42] to relay and integrative the sensory information of BP (Figure 11). The most importantly, the sex- and afferent-specific expression of Y_1_R and Y_2_R from this study would favor the explanation for the sex-difference in IbTX-mediated discharge profiles in the 1st-order BRNs [11, 36] of BRx pathway.

Although Y_1_R expression in tissue level of NG is slightly increased but statistical significance is not established between female and OVX model perhaps due to the smaller mass of ganglion tissue leading to a relatively large variation and supported by the notion that Y_1_R expression could be further downregulation after estrogen treatment. Whereas, in case of Y_2_R, the expression is identical between sexes but the OVX-mediated upregulation is restored with the treatment of estrogen, implying that OVX led Y_2_R upregulation would be explained as the compensatory mechanism at NG level to counteract elevated Y_1_R expression when lacking of estrogen. In addition, immunofluorescence showed that the Y_1_R expression is further downregulated while Y_2_R upregulation in HCN1-positive populations and together with the opposite pressor response mediated by Y_1_R and Y_2_R, the compensatory neuromodulation of NPY through its receptors in neurocontrol of circulation and BP regulation is sex-dimorphic and estrogen-dependent.

Taken together, we conclude that NPY would be a key player in either peripheral or central pathway in the regulation of blood pressure, and collaborative expression pattern between Y_1_R and Y_2_R at either NG or NTS level as well as an opposite pressor response of Y_1_R and Y_2_R would greatly impact on a sexual dimorphism of neurocontrol of circulation and BP.

## MATERIALS AND METHODS

An expanded methods section is available in the online-only data supplement.

### Arterial baroreflex sensitivity

Various doses of phenylephrine (PE) and sodium nitroprusside (SNP) were injected intravenously to measure the sex difference in baroreceptor sensitivity (BRS).

### Protein expression of Y_1_R and Y_2_R

Western Blot analysis was performed for testing relative expression of Y_1_R and Y_2_R.

### Immunohistochemical staining

Due to the afferent-specific expression, the antibody against for HCN1 was selected in this experiment as the fluorescent marker for myelinated afferents [16, 43], so, HCN1-positive and HCN-1-negative neurons were presumably classified as myelinated and unmyelinated afferents.

### Nodose ganglion microinjection of NPY and its receptor agonists

As described in the literature [44], after recording the baseline (before surgery) of blood pressure, the left side NG was exposed and 2 μl saline as the vehicle control was directly injected into tissue of ganglion using the specific designed needle to confirm the functional intact of Vagus and the baseline BP. In the following observation, NPY, Pro34 (Y_1_R agonist), and NPY13-36(Y_2_R agonist) were injected, respectively. The net changes in mean arterial pressure (MAP) were collected and analyzed by using the software of Labchart 7.

### Single-cell quantitative RT-PCR

In order to test the target mRNA examination in afferent-specific manner, qRT-PCR was carried out with identical procedures as previously described [16] in single-neurons identified by standard validation [13].

### Neuron afferent type identification

Afferent fiber types of isolated neurons were classified as myelinated A-, Ah-, and unmyelinated C-types according to electrophysiological and pharmacological validations [13] as well as morphological parameters [14]. The neurons from slice preparation were identified by afferent conduction velocity (CV) [45]. The afferent modality of baroreceptor of the 1st-order BRNs housed in nodose were also identified by the fluorescence [46].

## SUPPLEMENTARY MATERIALS FIGURES AND TABLES


