# Supplementary material for: Neuropeptide Y-mediated sex- and afferent-specific neurotransmissions contribute to sexual dimorphism of baroreflex afferent function
Source: Oncotarget. 2016 Sep 7;7(40):66135–48. doi: 10.18632/oncotarget.11880 (PMC5323221; doi:10.18632/oncotarget.11880)
Supplement: Supplementary file 1 [file oncotarget-07-66135-s001.pdf]

Neuropeptide Y-mediated sex- and afferent-specific neurotransmissions contribute to sexual dimorphism of baroreflex afferent function

SUPPLEMENTARY DATA

EXPANDED METHODS

Chemicals

17β-estradiol (17β-E2), BIBP3226 (a selective antagonist for Y1R), capsaicin (Cap, a TRPV1 agonist), and Iberiotoxin (IbTX, a selective blocker for large conductance of Ca^{2+}-activated-K^{+} channel, KCa1.1) were purchased from Sigma (St Louis, MO, USA); [Leu^{31}-Pro^{34}]-NPY (Pro34, a selective agonist for Y1R), NPY13-36 (a selective agonists for Y2R), BIIE0246 (a selective antagonist for Y2R), ω-Conotoxin GVIA (ω-CTX, a selective N-type Ca2+ channel blocker), and pertussis toxin (PTX, a G-protein coupling blocking agent) were purchased from Tocris (Ellisville, MO, USA). Stock solutions were stored at -20°C and diluted in bath solution right before experiments. In the experiment, drugs or toxins were applied through bath perfusion or microperfusion right on the patched neuron at flow rate no more than 1 ml/min.

Experimental animal

Age-matched adult male, ovaries intact (OVI) female and ovariectomized (OVX) female Sprague Dawley (SD) rats (12-14 weeks weighing 240-280g) were used in this study for molecular experiments, isolated neuron and Vagus-nodose slice preparations. Rats were directly purchased from Wei Tong Li Hua Experimental Animal Technology Co, Ltd, Beijing, China, with SPF grade and licensed under SCXK (Beijing) 2012–0001. All rats were maintained at the animal facility of the Second Affiliated Hospital of Harbin Medical University with a 12/12 hour light cycle for 3 days before they were used for experiments. All animal use protocols were pre-approved by the Institutional Animal Care and Use Committees of the School of Medical Science, Harbin Medical University, China.

Surgical ovariectomy

The surgery was performed following protocols described in details previously [1]. Briefly, anesthetized animals (combination of xylazine 10 mg/kg and ketamine 75 mg/kg) were placed in a lateral position, and both flanks were shaved and cleaned using chlorhexidine scrub and disinfected with 70% ethanol and povidone-iodine (7.5%). A 2.0-cm incision was made on the left lateral side along a line spanning from the 2nd to the 5th lumbar vertebra, using a scalpel blade. The left ovary and associated fat were located and externalized by gentle retraction. After removal of the ovary, the peritoneal cavity, muscle layers, and skin were closed successively with 4-0 absorbable sutures and then penicillin (80000 Units) was given via intramuscular injection. The same procedure was repeated for removal of the right ovary. After recovering from anesthesia, the animals were monitored for at least 30 min to ensure that there was no bleeding from the surgery, and then were returned to the animal facility. Four weeks after ovariectomy, a part of them were administrated 17β-estradiol (17β-E2) via subcutaneous injection (10 μg/kg, once daily) delivered in 200 μl sesame oil [2] for another 3 weeks, which were sacrificed for experimental use.

Arterial baroreflex sensitivity

The arterial was cannulated to connect the physiological pressure transducer (AD Instruments MLT 844, Norway), which was used for measuring the change of mean arterial blood pressure (MAP) and heart rate (HR) automatically at the same time. Phenylephrine (PE; Sigma Chemical) or sodium nitroprusside (SNP; Sigma Chemical) was injected by indwelling venous catheter to induce transient increase or decrease in BP with three different doses (2, 5, and 10 μg/kg) respectively. After the HR and arterial blood pressure responses to the first drug reached plateau, the second drug would be administrated. The calculation method of baroreflex sensitivity was described as Min Lin [3]. The maximal change of HR (ΔHR) and the value between the maximal MABP and the baseline MABP (ΔMABP) were recorded for all the groups. The averaged ratio of HR and MABP (ΔHR/ΔMABP) was designed as the symbol of BRS, which was applied to each dose of PE and SNP. All the data processing were applied by using the software of Labchart 7.

Measurement of serum NPY concentration

After animals were anaesthetized, the blood of the three group rats were collected immediately and allowed samples to clot at 4°C overnight before centrifugation for 20 minutes at 1000×g. Then the supernatant were collected

to measure the NPY concentration by using NPY enzyme-linked immunosorbent Assay Kit (Cloud-clone Corp., Houston, USA) according to the manufacturer's instructions.

Aortic depressor nerve (ADN) labeling

The fluorescent labeling of ADN with dye DiI was performed and the procedures were described as previously reported [4-6]. Briefly, after the anesthesia with the cocktail mentioned above and proper treatments of the skin on the surgical area, a ~2 cm incision was made under aseptic conditions along the left ventral side of the neck. A blunt dissection of the underlying musculature exposed the left carotid artery and surrounding nerve fibers. Under higher magnification, the left ADN, which exclusively contains baroreceptor fibers arising from the aortic arch, was identified. The ADN was separated from the Vagus and sympathetic nerves and placed in a 5 mm long sterile silicon trough. A few crystals of the lipophilic fluorescent dye DiI was placed on the ADN. The nerve, dye crystals and trough were coated with ~0.3 ml of a peripheral nerve encapsulant (Kwik-Sil, WPI). The area was rinsed with sterile saline and the skin was closed using vicryl suture. The animal fully recovered before returning to the animal facility. At least 4 weeks passed before the animal was considered fully healed and available for experimentation.

Western blot analysis of Y1R and Y2R protein

Each group consisted of ten animals that were euthanized with an excessive dose of pentobarbital sodium. Both sides of the nodose ganglions were removed immediately and frozen on dry ice. Ten pairs of tissue were collected for Western blots. The tissues were lysed by using lysis buffer (Beyotime Biotechnology Institute, China) containing 1% protease inhibitor solution (Beyotime). The lysate was centrifuged for 15 min to collect supernatant. The protein concentration was determined using BCA Protein Assay Kit (Beyotime Biotechnology Institute). The samples were boiled for 5 min, followed by loading on 12% SDS-PAGE gel (100 μg of protein, 15 μl per well) for electrophoresis using 110 V for 90 min. The protein on the gel was transferred onto NC membranes at 300 mA for 75 min, which was blocked by 5% nonfat dry milk diluted by PBS at room temperature for 3 hours. The membrane was probed with primary antibody (Y1 receptor rabbit polyclonal antibody, Alomone, 1:200 or Y2 receptor rabbit polyclonal antibody, Alomone, 1:200) at 4°C overnight and secondary antibody (goat anti-rabbit, Abcam, 1:8000) for 60 min at room temperature. GAPDH was used as the internal control. Bound bands were visualized and analyzed using Odyssey Infrared Imaging System (LI-COR Biosciences) and Odyssey v1.2 software.

Real-time quantitative PCR in tissue level

For SYBR Green RT-PCR, 4 SD rats were used to harvest total nodose ganglion mRNAs in each group. Total RNAs were extracted using the TRIzol® Reagent (Invitrogen) according to the manufacturer's instructions. The cDNAs were synthesized using the Reverse Transcription Kit (Applied Biosystems). Quantitative PCR reactions were run on an ABI 7500 fast Real-Rime PCR System (Applied Biosystems). The primers (Invitrogen) were used as follows: 5'-GGAGACCATGTGCAAACTGA-3' (forward) and 5'-CACCCTCTTGGGTTGATGAT-3' (reverse) for Y_1R; 5'-AGCCTTTCCACCCTGCTAAT -3' (forward) and 5'-CCTTCGCTGATGGTAATGGT-3' (reverse) for Y_2R. GAPDH was used as an internal control. The primer sequences for GAPDH were: 5'-AA GAAGGTGGTGAAGCAGGC -3' (forward) and 5'-TCC ACCACCCAGTTGCTGTA-3' (reverse). The $2^{-\Delta\Delta Ct}$ method was applied for the data analysis and the data were normalized and converted into relative mRNA expression.

For single-cell RT-PCR, after recording the AP for identifying the subtypes of VGNs as mentioned above, individual identified neuron was aspirated into a large-diameter pipette (3-7 μm) with ~2 μl of DEPC treated water in the tip and then immediately transferred into 5 μl of DEPC treated water at the bottom of the 0.2 ml EP tube, which was then stored at -80°C immediately for further analyze. SuperScript™ III CellsDirect cDNA Synthesis System (Invitrogen) was used to obtain cDNA. The other experimental conditions and analytic method are as the same with the tissue RT-PCR. The mRNA expression is based on the threshold cycles (Ct) and the appearance of GAPDH Ct proved the cells are successfully transferred into the EP tube.

Immunohistochemical analysis

Isolated one vagal ganglion of each group and section the embedded vagal ganglion into a thickness (8 μm) using the cryostat (LEICA cm 1850). Sections were placed onto glass slides and fixed with pre-cooled 4% paraformaldehyde for 10 min. Rinse the slides two times in phosphate buffered saline (PBS). Pour off the fixative and allow PBS to evaporate from the sections for 20 min at room temperature or stored at -80°C for later use. Sections were selected and rinsed in PBS for 3 min immediately, and incubated in PBS containing 1% BSA and 0.4% Triton X-100 (Sigma, St. Louis, MO, USA) for 1 h at 37°C. After blocking in 10% normal goat serum at 37°C for 2 h, sections were incubated with primary antibodies consisting Y1R (Alomone, 1:100) or Y2R

antibody (Alomone, 1:100) in PBS for 2 h, Subsequently further incubated with HCN1 (Abcam, 1:100) diluted in PBS from 594 rabbit and 488 mouse (Life Molecular Probe, 1:100) for 1 h at 37°C. Sections were then washed three final times in PBS briefly and coverslipped using anti-quenching agent with DAPI diluted by PBS. Imaging was under confocal microscope (Olympus Fluo-view 300).

Direct injection of NPY and its agonists into nodose ganglia

Rat were anesthetized (initial injection xylazine: ketamine 10: 75 mg/kg) and the femoral arterial was cannulated to connect the physiological pressure transducer (AD Instruments MLT 844, Norway), which was used to record the baseline arterial blood pressure before neck surgery. Rats were placed in a supine position and the neck was shaved and disinfected with 70% ethanol and povidone-iodine (7.5%). A 4.0 cm midline longitudinal cut was made and isolated one side of nodose ganglion under stereomicroscopy (×40) to make sure separate the Vagus nerve from the carotid, which was cleaned to avoid any connective tissue to reducing resistance when injecting (Supplementary Figure S1 NG injection A). During recordings, surgical stage of anesthesia was monitored by tail pinch, and if necessary, rats were re-injected with anesthetic (xylazine: ketamine 5: 75 mg/kg). After the surgery baseline MABP was stable, placed a clamp to apply slight tension on the Vagus nerve and punctured the nodose ganglion using a precision glass syringe (HAMILTON) affixed with a 30G half – inch stainless steel syringe needle with a 35° beveled tip with 2 μl grugs in saline. Rapid drop and recovery of blood pressure was detected if we have punctured vehicle into the nodose ganglion (Supplementary Figure 6A-top) and nerves were functionally intact. To testify that nodose ganglion was not punctured through it, NG was embedded after injected Evans blue and placed in a -80°C freezer for 1 hour before being sectioned into a thickness (20 μm) using the cryostat (LEICA cm 1850). Images were captured by scanning electron microscopy (Nikon, ECLIPSE 80) directly to observe the pinhole size (Supplementary Figure S1 NG injection B-D). Nodose injection recording were repeated to analysis the value of ΔMABP by using the software of Labchart 7 to detect the drugs effect.

Isolated neurons for electrophysiology

Isolation procedure was described in details previously [7-9]. Briefly, after both sides of nodose ganglia of female rat were surgically removed, they were cut into six pieces of each ganglion and then placed in cold nodose complete medium (NCM) composed of Mito™ + serum extender (v/v, 1:20, Collaborative Biomedical Products, Bedford, USA), Dulbecco's modified Eagle's medium F-12 (Gibco, Grand Island, USA) supplemented with 5% fetal bovine serum (HyClone, Logan, USA) and 1% penicillin-streptomycin-neomycin antibiotic mixture (PSN; Gibco, Grand Island, USA). The ganglia were transferred to an the 1st enzyme solution containing 20 units/ml of Papain (Worthington) in Earle's balance salt solution (Sigma, San Louis, USA) at 37°C for 20-22 min. The enzyme solution was replaced with the 2nd enzyme solution containing 2 mg/ml of Dispase II (Roche) and 1 mg/ml of Collagenase type-II (Worthington) in Earle's balance salt solution (Sigma) at 37 °C for additional 30-35 min depending upon the animal age. The enzyme solution was replaced with NCM containing 1.5 mg/ml albumin (Bovine, Sigma) and the ganglia were dissociated by trituration with fire-polished pipettes. Cell suspension was placed on poly-D-lysine (Sigma, San Louis, USA)-coated glass coverslips and cultured at 37°C moisture environment for at least 4 h before recording.

Whole-cell current- and voltage-clamp patch recordings [8, 9] were conducted using an Amplifier 200B or 700B (Axon Instruments, Union City, USA). Borosilicate glass pipettes (BF150-86-10; Sutter, Novato, USA) were pulled (P-97, Sutter, USA) and polished (F-83, Narishige, Japan) down to a resistance of 1.2-1.8 MΩ. Following correction for all offsets, a giga-ohm seal was formed and the pipette capacitance was compensated. Total cell capacitance (30-50 pF) and electrode access resistance (3-5 MΩ) were also compensated (60-80%). Recordings were low pass filtered to 10 KHz and digitized at 50 KHz. Experimental protocols, data collection, and preliminary analysis were performed using pCLAMP 10.3 and Digidata 1440A (Axon Instruments, Union City, USA). Corrections for liquid junction potentials were taken into consideration before final data analysis. The single and repetitive AP firings elicited by brief pulse and step depolarization current injects were collected, respectively, from each tested neurons for neuronal identification and discharge profile analysis before and after applications [9].

Vagus-nodose slice for electrophysiology

Slices of nodose ganglia with intact vagal axons were prepared in a manner previously described [8, 9]. Briefly, adult SD rats (250–300 g) of either gender were used for the slice preparation. The unrestrained rats were placed in an airtight induction chamber for inhalation of the anesthetic Metofane (Methoxyflurane, Schering-Plough Animal Health Corp, Kenilworth, NJ, USA). Upon lack of reflex response to tail pinch the animals were immediately sectioned at the mid-auxiliary region, preserving at least 2 cm of the Vagus nerve. The

nodose ganglia with attached Vagus were excised under stereomicroscopy (×40). The tissue was immediately placed in chilled (4°C) recording solution. Slicing exposed the interior of the ganglion capsule and the tissue was placed in a solution of Earle's balance salt solution (Sigma) containing type II Collagenase (1.0 mg/ml) at 37°C for 40 – 45 min followed by the solution containing Trypsin-3X (5 mg/ml) for another 20 – 22 min. The tissue was moved to the bath perfusion chamber and allowed to recover for 1 h prior to recording. The identical whole-cell patch techniques were used as described above. After tightened seal, electric evoked currents by directly stimulation of Vagus [10] was collected under cell-attach configuration in AP bath solution with peptide solution for Ca^{2+} current recording, and neuron type could be identified upon the afferent fiber CV. And then whole-cell Ca^{2+} currents could be collected by completely bath perfusion with Ca^{2+} current recording solution. Cell was held at −100 mV and voltage was stepped from−80 mV to +30 mV for 400 ms with 10 mV increments and 1 s interval between steps.

Action potential and Ca^{2+} current recordings

Whole-cell patch [8, 9] was conducted using an Amplifier 200B or 700B (Axon Instruments, Union City, USA). Borosilicate glass pipettes (BF150-86-10; Sutter, Novato, USA) were pulled (P-97, Sutter, USA) and polished (F-83, Narishige, Japan) down to a resistance of 1.2-1.8 MΩ. Following correction for all offsets, a giga-ohm seal was formed and the pipette capacitance was compensated. Total cell capacitance (30-50 pF) and electrode access resistance (3-5 MΩ) were also compensated (60-80%). For isolated neuron identification and testing the effect of Y1R or Y2R activation on discharge profiles, a single AP and repetitive discharge were elicited under current-clamp mode by a brief current pulse and step depolarization. After control recordings, drug was applied through bath perfusion at flow rate no more than 1 ml/min and induced change in membrane potential was recorded. Recordings were low pass filtered to 10 KHz and digitized at 50 KHz. Experimental protocols, data collection, and preliminary analysis were performed using pCLAMP 10.3 and Digidata 1440A (Axon Instruments, Union City, USA). Corrections for liquid junction potentials were taken into consideration before final data analysis. For Ca^{2+} current recording, slice preparation was employed and the afferent fiber types were firstly identified by conduction velocity (CV) in cell-attached mode with AP recording solution followed by completely perfusion with extracellular solution for Ca^{2+} recording, and then ruptured the membrane and Ca^{2+} current recording [8, 10].

Neuron afferent type identification

According to our electrophysiological validations [9] and morphological parameters [11] for isolated neuron identification, those neurons showing AP firing threshold (APFT) below -40 mV, maximal upstroke velocity (UV_{MAX}) over 300 mV/ms, half duration of AP (APD_{50}) less than 2.0 ms with a repolarization hump are classified as myelinated Ah-types; whereas, myelinated A-types show much narrow APD_{50} with no hump compared with Ah-types. Those neurons showing APFT at -35 mV or less negative, UV_{MAX} well below 250 mV/ms, and APD_{50} over 2.0 ms with significant repolarization hump are identified as unmyelinated C-types. Additionally, morphological parameters would help to closely estimate neuron-type before patch recordings.

Recording solutions

For action potential (AP) recordings (isolated neuron): an extracellular solution containing (in mM): 137 NaCl, 5.4 KCl, 1.0 $MgCl_2$, 2.0 $CaCl_2$, 10 glucose, and 10 HEPES with pH adjusted to 7.35 using 1.0 N NaOH. The pipette solution for the recordings contained (in mM): 140 potassium aspartate, 3.0 $MgCl_2$, 4.0 BAPTA-K, 10 HEPES, and 0.25 $CaCl_2$ for a final buffered intracellular calcium concentration of 100 nM with pH adjusted to 7.3 using 1.0 N KOH. Prior to recording, 2.0 mM Mg-ATP was added to the pipette solution. Osmolarities of extracellular and pipette solutions were adjusted using D-mannitol (Sigma) to 310 and 295 mOsm, respectively. All recordings were performed at room temperature (22-23°C).

For whole-cell Ca^{2+} current recordings (nodose slice): Pipette solution for measuring whole-cell Ca2+ currents contained (in mM) 124.0 CsCl, 5.0 $MgCl_2$, 10.0 glucose, 10.0 HEPES and 10 HEPES (pH 7.25), whereas the bath solution was composed of (in mM) 140.0 TEA-Cl, 5.0 4-AP, 15.0 HEPES, 20.0 glucose, and 2.0 $CaCl_2$ (pH 7.4). Osmolarities of extracellular and pipette solutions were adjusted using D-mannitol (Sigma) to 310 and 295 mOsm, respectively. All recordings were performed at room temperature (22-23°C).

For AP and EPSC recordings (NTS brain slice): an artificial CSF (ACSF) composed of the following (in mm): 125 NaCl, 3 KCl, 1.2 KH_2PO_4, 1.2 $MgSO_4$, 25 $NaHCO_3$, 10 dextrose, and 2.0 $CaCl_2$. An electrodes were filled with a solution composed of the following (in mm): 10 NaCl, 110 K-gluconate, 20 KOH, 11 EGTA, 1 $CaCl_2$, 2 $MgCl_2$, 10 HEPES, 1 Na-ATP, and 0.1 Na-GTP, pH 7.3 (295 mOsm). All recordings were performed at near body temperature (35-36°C).

Data analysis

Clampfit (Molecular Devices; Sunnyvale, USA) was used for initial data readings for statistical analysis (Excel; Microsoft, Northampton, USA) and trace filtering and data reduction processes for data graphing (Origin; Microsoft, Northampton, USA). Statistical differences between expression levels were determined by either a Student's *t*-test or an ANOVA. A paired *t*-test was used to compare the difference before and after treatment and average data were presented as mean ± SD. Fluorescence intensity or density was measured by Image-pro plus 5.0 (Media Cybernetics, USA). The value equal or less than 0.05 was considered as significantly difference compared with before test.

REFERENCES

1. Qiao GF, Qian Z, Sun HL, Xu WX, Yan ZY, Liu Y, et al. Remodeling of hyperpolarization-activated current, Ih, in Ah-type visceral ganglion neurons following ovariectomy in adult rats. PLoS One. 2013;8:e71184.
2. Lim DW, Kim JG, Lee Y, Cha SH, Kim YT. Preventive effects of Eleutherococcus senticosus bark extract in OVX-induced osteoporosis in rats. Molecules. 2013;18:7998-8008.
3. Lin M, Liu R, Gozal D, Wead WB, Chapleau MW, Wurster R, et al. Chronic intermittent hypoxia impairs baroreflex control of heart rate but enhances heart rate responses to vagal efferent stimulation in anesthetized mice. Am J Physiol Heart Circ Physiol. 2007;293:H997-1006.
4. Doyle MW, Bailey TW, Jin YH, Appleyard SM, Low MJ, Andresen MC. Strategies for cellular identification in nucleus tractus solitarius slices. J Neurosci Methods. 2004;137:37-48.
5. Li BY, Qiao GF, Feng B, Zhao RB, Lu YJ, Schild JH. Electrophysiological and neuroanatomical evidence of sexual dimorphism in aortic baroreceptor and vagal afferents in rat. Am J Physiol Regul Integr Comp Physiol. 2008;295:R1301-10.
6. Han LM, Ban T, Liu Y, Yuan M, He JL, Wen X, et al. Hyperpolarization-activated current-mediated slow afterhyperpolarization in myelinated Ah-type of baroreceptor neurons isolated from adult female rats. Int J Cardiol. 2014;172:e106-8.
7. Li BY, Schild JH. Comparisons of somatic action potentials from dispersed and intact rat nodose ganglia using patch-clamp technique. Acta Pharmacol Sin. 2002;23:481-9.
8. Li BY, Schild JH. Patch clamp electrophysiology in nodose ganglia of adult rat. J Neurosci Methods. 2002;115:157-67.
9. Li BY, Schild JH. Electrophysiological and pharmacological validation of vagal afferent fiber type of neurons enzymatically isolated from rat nodose ganglia. J Neurosci Methods. 2007;164:75-85.
10. Huo R, Xu WX, Han LM, He JL, Liu Y, Lu XL, et al. Fine tuning of calcium on membrane excitation of baroreceptor neurons in rats. Int J Cardiol. 2014;174:883-7.
11. Lu XL, Xu WX, Yan ZY, Qian Z, Xu B, Liu Y, et al. Subtype identification in acutely dissociated rat nodose ganglion neurons based on morphologic parameters. Int J Biol Sci. 2013;9:716-27.
12. Liu Y, Zhou JY, Zhou YH, Wu D, He JL, Han LM, et al. Unique Expression of Angiotensin Type-2 Receptor in Sex-Specific Distribution of Myelinated Ah-Type Baroreceptor Neuron Contributing to Sex-Dimorphic Neurocontrol of Circulation. Hypertension. 2016;67:783-91.

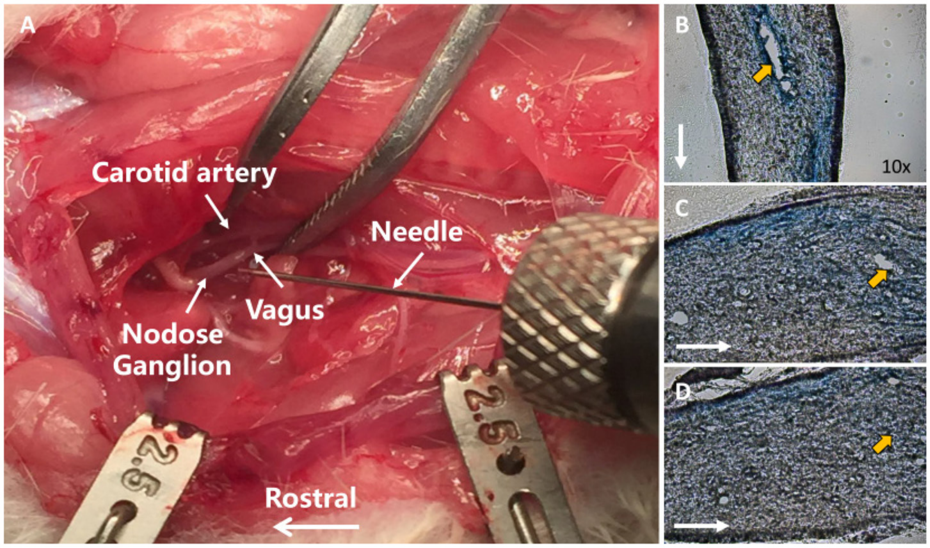

Supplementary Figure S1: Exposed nodose ganglia before microinjection. A. Separate the Vagus nerve from the carotid to expose the nodose ganglion under stereomicroscopy (x40). The arrow points the NG position. **B–D.** Microscopic identification of NG injection(x10). Yellow arrow points the pinhole position and certify not to puncture through it.

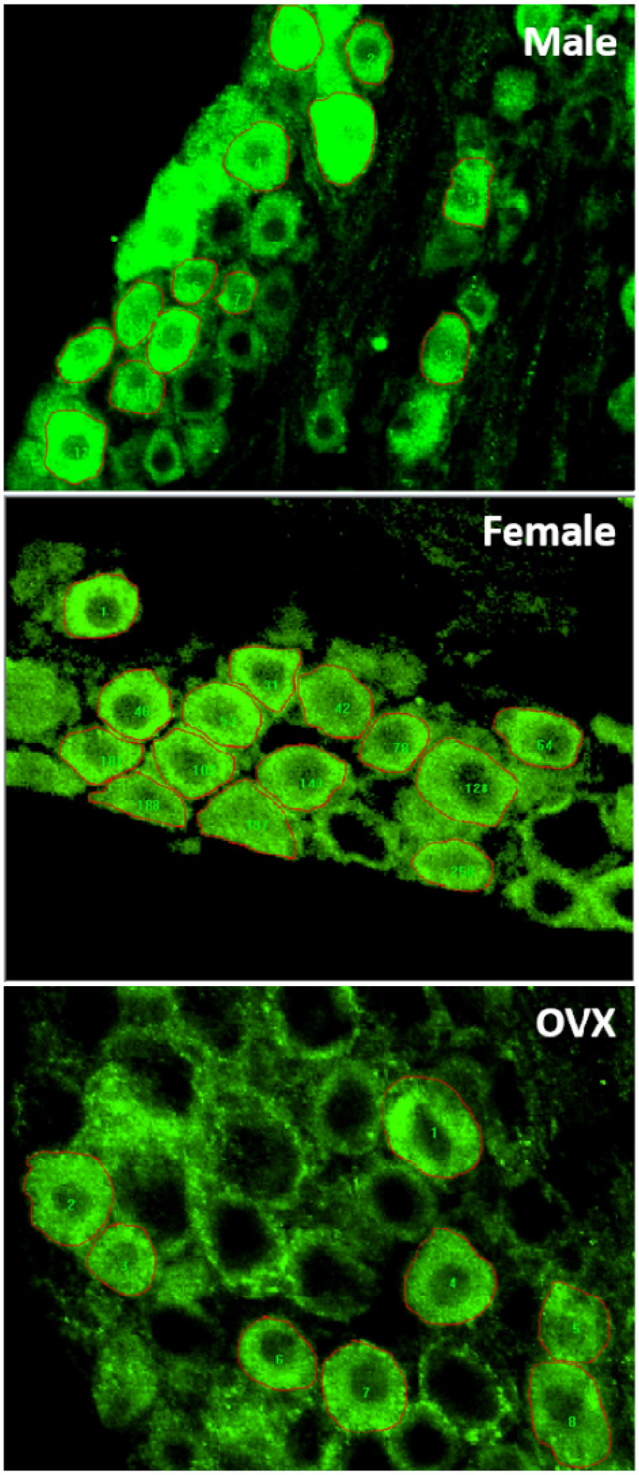

Supplementary Figure S2: Immunohistochemistry Analysis Methods Images were analyzed using Image-pro plus that was described in a manner previously [12]. Briefly, keeping the same background to distinguish the positive and negative objects, selected the Irregular Area of Interest (AOI) tool to manually outline objects, which encompassed the colors and holes. Measuremenu inclued the requested measurements of all the recorded objects, which can be viewed from the data sheet.

Supplementary Table S1: Quantified immunofluorescent intensity and comparison analysis for Y1R and Y2R

Group	HCN1-positive	*n*	HCN1-negative	*n*
	**Y1R**			
Male	205.3±29.0	13	90.3±32.7	9
Female	184.8±13.5*	14	70.4±15.9	11
OVX	152.1±10.6##	8	67.1±11.7	8
	**Y2R**			
Male	126.8±38.3	12	53.9±8.4	11
Female	126.0±15.1	11	62.4±5.7*	8
OVX	170.4±39.7##	12	52.5±13.6	8

*P < 0.05 and **P < 0.01 *vs.* male and ##P < 0.01 *vs.* female.

Supplementary Table S2: Effects of Y1R or Y2R activation on AP and *I*Ca in identified C-BRNs, EPSC profiles in identified C-NTS neurons

	NG: C-BRNs			
Parameter	APD_{50}	DV_{MAX}	APFF	N-type I_{Ca}
Control	2.76±0.6	51.4±9.2	1.26±0.4	2.06±0.7
Pro34	3.07±0.5*	41.6±6.8*	6.02±2.4**	1.58±0.6**
Control	2.84±0.4	49.3±7.6	1.63±1.8	2.24±0.6
NPY13-36	3.14±0.6*	36.7±5.7*	5.88±1.7**	1.71±0.5**
n = 9-11	*P < 0.05 and **P < 0.01 vs control			
